# Identification of specific calcitonin-like receptor residues important for calcitonin gene-related peptide high affinity binding

**DOI:** 10.1186/1471-2210-6-9

**Published:** 2006-06-15

**Authors:** Sugato Banerjee, Janel Evanson, Erik Harris, Stephen L Lowe, Kathryn A Thomasson, James E Porter

**Affiliations:** 1 Department of Pharmacology, Physiology & Therapeutics, School of Medicine & Health Sciences, University of North Dakota, Grand Forks, ND 58202-9037, USA; 2 Department of Chemistry, University of North Dakota, Grand Forks, ND 58202-9024, USA

## Abstract

**Background:**

Calcitonin gene-related peptide (CGRP) is a vasoactive neuropeptide whose biological activity has potential therapeutic value for many vascular related diseases. CGRP is a 37 amino acid neuropeptide that signals through a G protein-coupled receptor belonging to the secretin receptor family. Previous studies on the calcitonin-like receptor (CLR), which requires co-expression of the receptor-activity-modifying protein-1 (RAMP1) to function as a CGRP receptor, have shown an 18 amino acid N-terminus sequence important for binding CGRP. Moreover, several investigations have recognized the C-terminal amidated phenylalanine (F37) of CGRP as essential for docking to the mature receptor. Therefore, we hypothesize that hydrophobic amino acids within the previously characterized 18 amino acid CLR N-terminus domain are important binding contacts for the C-terminal phenylalaninamide of CGRP.

**Results:**

Two leucine residues within this previously characterized CLR N-terminus domain, when mutated to alanine and expressed on HEK293T cells stably transfected with RAMP1, demonstrated a significantly decreased binding affinity for CGRP compared to wild type receptor. Additional decreases in binding affinity for CGRP were not found when both leucine mutations were expressed in the same CLR construct. Decreased binding characteristic of these leucine mutant receptors was observed for all CGRP ligands tested that contained the necessary amidated phenylalanine at their C-terminus. However, there was no difference in the potency of CGRP to increase cAMP production by these leucine mutant receptors when compared to wild type CLR, consistent with the notion that the neuropeptide C-terminal F37 is important for docking but not activation of the receptor. This observation was conserved when modified CGRP ligands lacking the amidated F37 demonstrated similar potencies to generate cAMP at both wild type and mutant CLRs. Furthermore, these modified CGRP ligands displayed a significant but similar loss of binding for all leucine mutant and wild type CLR because the important receptor contact on the neuropeptide was missing in all experimental situations.

**Conclusion:**

These results are consistent with previous structure-function investigations of the neuropeptide and are the first to propose specific CLR binding contacts for the amidated F37 of CGRP that are important for docking but not activation of the mature CGRP receptor.

## Background

Calcitonin gene-related peptide (CGRP) released from sensory fibers originating in the trigeminal ganglia [1] or the adventitial-medial border of arteries supplying the heart [2] causes a potent, efficacious and long-lasting dilation of cerebral and coronary vessels, respectively [3,4]. Vasodilatation of cerebral arteries by CGRP released from trigeminal fibers is implicated in the pathogenesis of migraine headaches [5]. Conversely, vasodilatation by CGRP released in the peripheral vasculature might be of considerable importance to patients suffering from hypertension and/or heart disease [6,7]. For example, it has been demonstrated that CGRP is essential for myocardial protection, during reperfusion injury, either in ischemic preconditioning or in heat stress-induced cardioprotection [8]. However, lack of small molecule non-peptide CGRP receptor agonists or antagonists limit the essential investigations that would assess the role of this neuropeptide in vascular related diseases.

Structure-function relationships of CGRP have been extensively studied and are highlighted in a review by Wimalawansa [9]. Briefly, this 37 amino acid neuropeptide can be divided into two distinct domains based upon its binding and agonistic properties for the receptor. The first N-terminal residues (1–7) have been shown to be essential for receptor activation [10]. Specifically, loss of the disulfide bridge connecting amino acids 2 and 7 negates all biological activity of the endogenous neuropeptide [11]. However, deleting this portion of the CGRP molecule does not appear to be critical for receptor affinity [12]. Conversely, the N-terminal truncated peptide, CGRP(8–37), is an effective CGRP receptor antagonist having a high affinity for the receptor but devoid of any biological activity in itself [13]. More specifically, the amidated C-terminal phenylalanine at position 37 of CGRP has been shown to be necessary for receptor interaction since deletion of this peptide residue or loss of the C-terminal amine results in a significant deficiency of high affinity binding [14,15]. This affinity loss of *des*-F37-CGRP for the native receptor when compared to the endogenous neuropeptide is not due to disruption of the ligand structure [16]. Instead, it is postulated that the C-terminal phenylalaninamide of the ligand directly interacts with the mature CGRP receptor in order for this high affinity binding to occur. Moreover, conservative substitution of F37 for tyrosine, which adds only one hydroxyl group to the phenol ring, again causes a significant loss of binding affinity, supporting the idea that CGRP-F37 is essential for high affinity interaction with the mature receptor protein [17]. In addition, an α-helical configuration incorporating residues 8–18 and β-sheet structures related to amino acids 18–21 and 32–35 are also thought to contribute to the high affinity receptor antagonist profile of CGRP(8–37) [18,19]. However, there is no information in the literature regarding specific interactions of the receptor with these identified ligand domains, particularly the C-terminus phenylalaninamide of CGRP.

The receptor for CGRP is a heptahelical membrane protein that belongs to the group B family of G protein-coupled receptors (GPCR). This GPCR family is distinct from the well characterized group A rhodopsin family of GPCRs in the fact that group B members have long extracellular N-terminus domains and utilize moderately sized peptides as their endogenous agonists [20]. The human clone for this receptor has been identified and named the calcitonin-like receptor (CLR) protein based on sequence identity to the calcitonin receptor [21]. However, transient mammalian cell expression of the human CLR clone could not be achieved until identification of a single transmembrane protein family called receptor-activity-modifying proteins (RAMPs) that are essential for trafficking mature CLR proteins to the cell membrane surface [22]. In this study, heterodimerization of CLR-RAMP1 on the membrane surface was shown to exhibit the pharmacological profile of an endogenously expressed CGRP receptor. Conversely, interaction of CLR with RAMP2 or 3 on the membrane surface demonstrated the pharmacological receptor profile for a related peptide, adrenomedullin (AM). Therefore, cell specific expression of distinctive RAMPs with the CLR imparts selective pharmacological receptor profiles for related peptide families.

**Figure 1:**
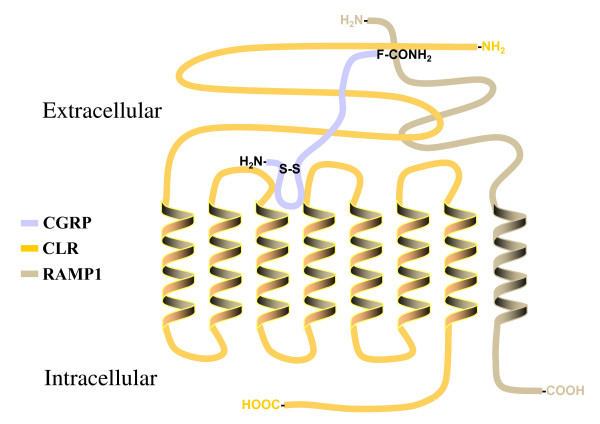
**Postulated interactions of specific CGRP domains with the CLR-RAMP1 heterodimer**. Co-expression of the CLR with RAMP1 forms a mature CGRP receptor on the cell membrane surface. The characteristically long extracellular domains of the CLR-RAMP heterodimer create a high affinity binding pocket important for docking the C-terminal phenylalaninamide of CGRP. This interaction presents the CGRP N-terminus domain, essential for biological activity, to a "classical" agonist binding pocket formed in part by the transmembrane α-helices of the CLR-RAMP1 heterodimer.

Structure-function studies of group B GPCR families have documented important interactions of the characteristically long N-terminal domain with their endogenous peptide ligands [23,24]. Recent evidence has also addressed the importance of the CLR N-terminal domain for docking with the endogenous neuropeptide. For example, a purified CLR N-terminal domain overexpressed in *E. coli *was able to specifically bind ^125^I-CGRP and could displace this same radiolabeled peptide from a membrane preparation containing native CGRP receptors [25]. This observation supports previous structure-function analysis of CGRP that describe two separate peptide domains essential for binding and agonism [11,15]. A model illustrating this interaction of specific CGRP domains with the CLR-RAMP1 heterodimer (*i.e*., mature CGRP receptor) is presented in figure 1.

Specific to the CLR, deletion of an 18 amino acid region of the receptor N-terminal domain resulted in a significant loss of ^125^I-CGRP binding when co-expressed in mammalian cells with RAMP1 [26]. However, this CLR deletion mutant was able to generate cAMP in a concentration-dependent manner with increasing amounts of CGRP. For our investigation, we considered whether the essential C-terminus phenylalaninamide of CGRP was contributing to high affinity neuropeptide binding by directly interacting with hydrophobic amino acids found within this deleted CLR N-terminal domain. Therefore, we used site-directed mutagenesis techniques to change these hydrophobic CLR residues to alanine, then characterized the binding and signaling properties of these transiently expressed CLR mutants for the endogenous or modified neuropeptides, comparing the results to wild type receptor. In this study, we identify two specific leucine residues within this N-terminal domain of the CLR that significantly contribute to the high affinity binding of CGRP without appreciably affecting receptor function.

## Results

### Competitive radioligand binding properties of CGRP and modified peptide ligands for wild type and mutant CLRs

**Figure 2:**
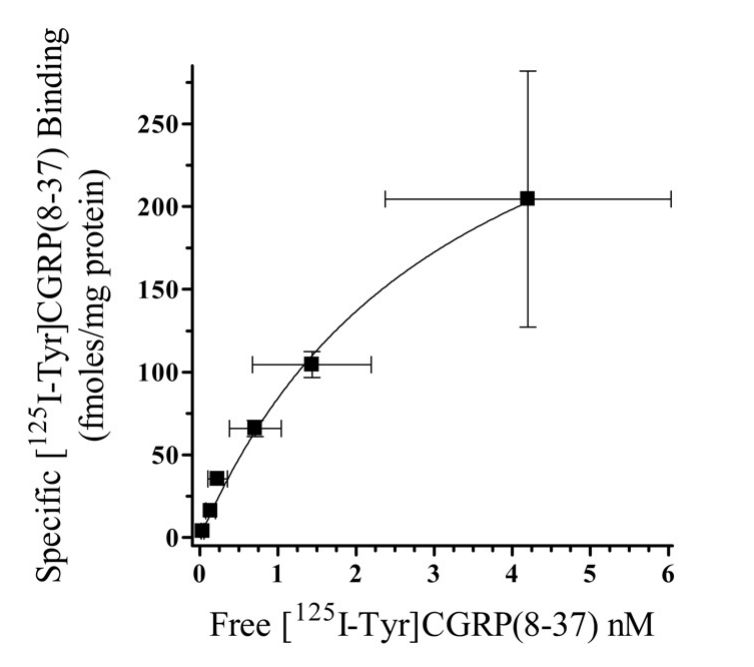
**Saturation binding characteristics of [^125^I-Tyr]CGRP(8–37)**. Increasing amounts of [^125^I-Tyr]CGRP(8–37) were used on a crude HEK293T-RAMP1 membrane preparation that had been transiently transfected with wild type CLR, in the absence and presence of 1μM CGRP to determine total and nonspecific binding, respectively. Non-linear regression analysis was used to best fit a specific binding curve to each individual binding experiment. From this best fit curve an equilibrium dissociation constant (K_d_) for the peptide radioligand and total number of specific binding sites (B_max_) were estimated. The K_d _of [^125^I-Tyr]CGRP(8–37) was calculated to be 0.9 ± 0.2 nM with a B_max _estimated at 285 ± 206 fmols/mg protein. The data presented represents the mean ± S.E. for *n *= 3 experiments performed in duplicate.

Previous investigations have identified [^125^I-Tyr]CGRP(8–37) as a selective high affinity radioligand for mature CGRP receptors (*i.e*., CLR-RAMP1 heterodimers) endogenously expressed in rat brain and peripheral tissues [27]. Therefore, we used this same radiochemical to recognize mature CGRP binding sites on HEK293T-RAMP1 cells that had been transiently transfected with mutant and wild type CLRs. Crude membrane preparations from these transfected HEK293T-RAMP1 cells were used in saturation binding experiments with increasing concentrations of [^125^I-Tyr]CGRP(8–37) in the absence or presence of 1μM CGRP to define nonspecific binding. Preliminary studies using non-transfected HEK293T-RAMP1 membranes or membranes isolated from cells transiently transfected with empty vector demonstrated no specific binding with increasing concentrations of the radioligand (data not shown). Conversely, HEK293T-RAMP1 membranes transiently expressing the wild type CLR demonstrated a saturable rise in specific binding as a result of increasing [^125^I-Tyr]CGRP(8–37) concentrations (Fig 2). The equilibrium dissociation constant of the radioligand (K_d_) and the maximal specific binding of [^125^I-Tyr]CGRP(8–37) (B_max_) calculated from non-linear regression analysis was 0.9 ± 0.2 nM and 285 ± 206 fmols/mg of wild type membrane protein, respectively (*n *= 3). This K_d _value is similar to affinity constants calculated for this radioligand when used in other investigations to identify endogenous CGRP receptor binding sites [27]. Analogous saturation binding experiments were performed for the L24A, L34A and L24A,L34A CLR double mutant transiently expressed individually on HEK293T-RAMP1 membranes with an approximate 10-fold decrease in the [^125^I-Tyr]CGRP(8–37) K_d _value calculated for all receptor mutations (Table 1). These K_d _values were used to calculate equilibrium dissociation constants (K_i_) of competing CGRP receptor active ligands for transiently expressed wild type and mutant CLR-RAMP1 heterodimers using the method of Cheng and Prusoff [28].

**Table 1:** Comparisons of K_d _and K_i _values to previously published equilibrium dissociation constants for endogenous and modified CGRP ligands (in nM).

Peptide Ligand	Wild Type	Reported Wild Type	L24A	L34A	L24A,L34A
[^125^I-Tyr]CGRP(8–37)	0.9 ± 0.2	0.2^*a*^	9.0	9.0	9.0
CGRP	1.4 ± 0.3	0.4_H_, 32_L _^*a*^	8.9 ± 3.1	10.8 ± 2.2	9.3 ± 2.8
AcCGRP(19–37)	227 ± 86	933^*b*^	3000 ± 1400	4600 ± 1200	2000 ± 1000
CGRP(1–36)	> 10,000	n.d.	> 10,000	> 10,000	> 10,000
CGRP(1–19)	> 10,000	n.d.	> 10,000	> 10,000	> 10,000

^*a*^Van Rossum *et al*., 1994; ^*b*^Rovero *et al*., 1992;

H and L represent high and low binding affinity values respectively calculated from a complex curve fitting model of the data points.

**Figure 3:**
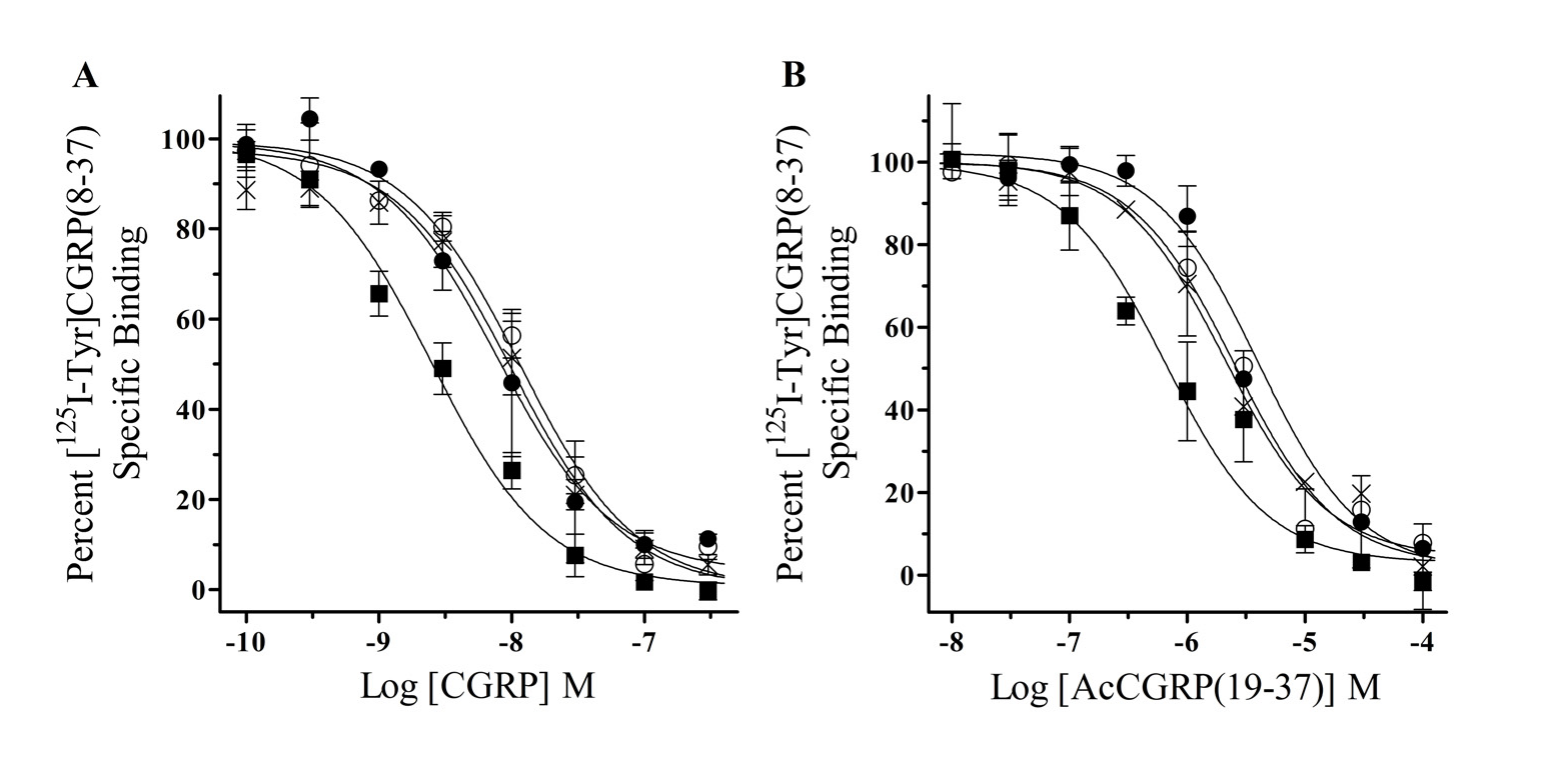
**Competition binding characteristics of CGRP and AcCGRP(19–37) for wild type and mutant CLRs**. Increasing amounts of (**A**) CGRP or (**B**) AcCGRP(19–37) were used to compete for specific [^125^I-Tyr]CGRP(8–37) binding sites on crude HEK293T-RAMP1 membrane preparations transiently transfected, individually with wild type (■), L24A (●), L34A (○) or L24A,L34A (x) CLR mutations. Non-linear regression analysis was used to best fit a sigmoidal curve from the data points of each individual competition binding experiment. From this best fit curve the concentration of competing peptide need to displace 50% of specific radioligand binding (IC_50_) was estimated and used to calculate the equilibrium dissociation constant (K_i_) of unlabled peptide for each CLR. The calculated K_i _values of the competing peptides for the L24A, L34A, or L24A,L34A CLR mutants were significantly different (*P *< .05) from the wild type receptor and are displayed in Table 1. The data presented represents the mean ± S.E. for *n *= 3–5 experiments performed in duplicate.

Increasing concentrations of CGRP or the N-terminally acetylated peptide fragment, AcCGRP(19–37) were used to inhibit specific [^125^I-Tyr]CGRP(8–37) binding to membranes prepared from HEK293T-RAMP1 cells transiently transfected with wild type or mutant CLRs. Specific [^125^I-Tyr]CGRP(8–37) binding was plotted versus increasing concentrations of the endogenous or modified neuropeptide and a competition curve for each membrane preparation was generated using non-linear regression analysis (Fig 3). When the IC_50 _of these curves were used to calculate the K_i _of CGRP for these transiently expressed membrane proteins (Fig 3A), there was a significant 6 to 8-fold decrease in affinity values for all mutant receptors (L24A = 8.9 ± 3.1 nM, *n *= 3; L34A = 10.8 ± 2.2 nM, *n *= 4; L24A,L34A = 9.3 ± 2.8 nM, *n *= 3) when compared to wild type CLR (1.4 ± 0.3 nM, *n *= 5). Likewise, the K_i _of AcCGRP(19–37) calculated from competition binding experiments (Fig 3B) using transiently transfected wild type CLR membranes (227 ± 86 nM, *n *= 3) was similar to the equilibrium dissociation constant (933 ± 421 nM, *n *= 3) estimated from functional characterization of endogenous CGRP receptors expressed in the guinea pig atria [29]. However, there was a significant 10 to 20-fold decrease in the K_i _values of AcCGRP(19–37) for the L24A (3.0 ± 1.4μM, *n *= 3), L34A (4.6 ± 1.2μM, *n *= 3) and L24A,L34A (2.0 ± 1.0μM, *n *= 5) double mutant CLR when compared to wild type (Fig 3B). Since the amidated F37 is a common residue not only of the radioligand, but also the two competitive peptides, similarities in binding affinity loss lend support to our hypothesis that hydrophobic N-terminus CLR amino acids are docking with the C-terminal phenylalaninamide of the neuropeptide. Furthermore, there were no significant differences in affinity values of the endogenous or modified competitive peptides for single and double leucine CLR mutations, suggesting that no synergistic binding relationship between CGRP-F37 and these hydrophobic CLR residues is occurring.

To specifically test this hypothesis we used modified peptide ligands, which lacked the C-terminus phenylalanine in additional competitive radioligand binding experiments. There was a significant affinity loss of the C-terminal amidated ligand, CGRP(1–36), for the wild type CLR transiently expressed on HEK293T-RAMP1 membranes when compared to the K_i _of CGRP for the same receptor (Table 1). However, there were no differences in CGRP(1–36) affinity values for L24A, L34A and L24A,L34A receptor mutants when compared to wild type CLR. To confirm these observations we utilized another truncated CGRP fragment, which was not amidated at the carboxyl terminus. Similar to results using CGRP(1–36), there was a significant loss of CGRP(1–19) affinity for the wild type CLR when compared to the endogenous neuropeptide at the same receptor (Table 1). Nevertheless, this CGRP(1–19) affinity for the wild type receptor was analogous to values calculated for all leucine CLR mutations. These results emphasized the importance of the C-terminal F37 as an important binding contact for the wild type CLR (12). In addition, the lack of CGRP(1–36) or CGRP(1–19) affinity value changes for mutant compared to wild type CLRs, suggests that N-terminus leucines at positions 24 and 34 on the receptor might be interacting with F37 of the endogenous neuropeptide.

### Signaling properties of stimulated wild type and mutant CLRs using CGRP or modified receptor active peptide ligands

**Figure 4:**
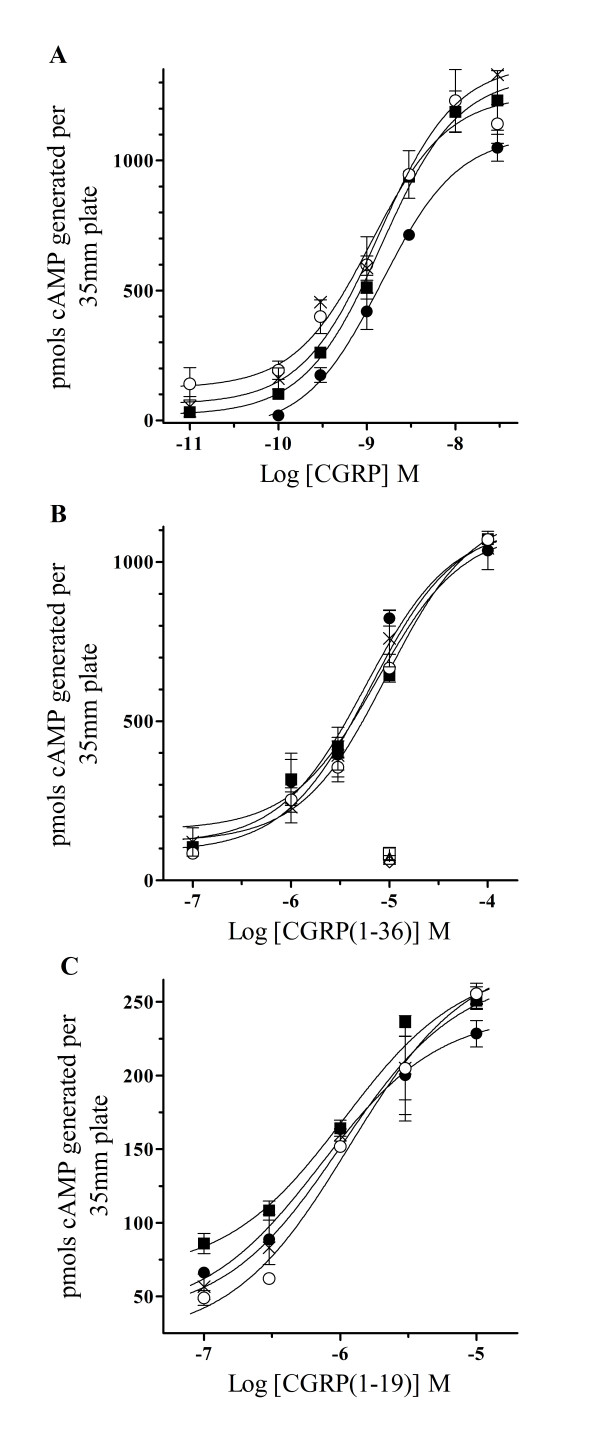
**cAMP production of activated wild type and CLR mutants using endogenous or truncated CGRP ligands**. Increasing amounts of (**A**) CGRP, (**B**) CGRP(1–36) or (**C**) CGRP(1–19) were used to stimulate confluent HEK293T-RAMP1 cells that had been transiently transfected, individually with wild type (■), L24A (●), L34A (○) or L24A,L34A (x) CLR mutations. (**B**) Other groups of HEK-RAMP1 cells transiently transfected, individually with wild type (□), L24A (△), L34A (+) or L24A,L34A (◊) CLR mutations were treated with 1μM of the CGRP receptor antagonist, CGRP(8–37), for 30 min prior to the addition of 10μM CGRP(1–36). After 30 min all cells were lysed and the amount of cAMP generated was quantified using a radioimmunoassay according to the manufactures protocol (Amersham). Non-linear regression analysis was used to best fit a sigmoidal curve from the data points of each individual experiment. From this best fit curve a concentration of peptide agonist that produced 50% of the maximal cAMP response (EC_50_) was estimated for each CLR. The calculated EC_50 _values of all peptides to increase cAMP in HEK293T-RAMP1 cells transiently transfected with L24A, L34A or L24A,L34A CLR mutations were no different from values calculated for the wild type receptor and are displayed in table 2. The data presented represents the mean ± S.E. for *n *= 3 experiments performed in duplicate.

CGRP-F37 is hypothesized to be an important binding contact for hydrophobic N-terminus residues on the CLR. However, it is important to show if this ligand-receptor contact is also central to activation mechanisms of the mature membrane protein. Neuropeptide activation of endogenously expressed CLR-RAMP1 heterodimers has been shown to generate increases in cAMP [9]. Therefore, we examined the characteristics of CGRP and other modified peptide agonists to generate cAMP in HEK293T-RAMP1 cells that had been transiently transfected with wild type and leucine CLR mutations in order to test the importance of these residues for receptor activation. Preliminary studies using non-transfected HEK293T-RAMP1 cells or cells transiently transfected with empty vector showed no production of cAMP after 30 min incubation at 37°C with 10 nM CGRP (data not shown). Conversely, increasing concentrations of CGRP generated rising amounts of cAMP in HEK293T-RAMP1 cells transiently expressing wild type or mutant CLRs (Fig 4A). The potency of CGRP to generate cAMP in these cells transfected with the wild type receptor (1.6 ± 0.1 nM, *n *= 3) was not significantly different from HEK293T-RAMP1 cells expressing the L24A (1.5 ± 0.4 nM, *n *= 3), L34A (2.0 ± 0.5 nM, *n *= 3) or double L24A,L34A (1.3 ± 0.2 nM, *n *= 3) CLR mutants. This suggests that modification of these N-terminus CLR leucines to alanine, individually or in tandem, does not affect the neuropeptide initiated signaling of the membrane protein.

**Table 2:** Potency (EC_50_) values of endogenous and modified CGRP ligands to generate cAMP in transiently transfected HEK293T-RAMP1 cells (in nM).

Peptide Agonist	Wild Type	L24A	L34A	L24A,L34A
CGRP	1.6 ± 0.1	1.5 ± 0.4	2.0 ± 0.5	1.3 ± 0.2
CGRP(1–36)	4300 ± 800	3400 ± 700	5600 ± 800	4200 ± 1900
CGRP(1–19)	2100 ± 600	2400 ± 200	2200 ± 600	1600 ± 200

To support functional results observed for CGRP mediated generation of cAMP through wild type and CLR mutants, we used the truncated peptide, CGRP(1–36), which lacked F37 but was still amidated at the C-terminus. Increasing amounts of CGRP(1–36) was able to generate cAMP in HEK293T-RAMP1 cells transiently expressing the wild type CLR in a concentration-dependent manner (Fig 4B) The EC_50 _of CGRP(1–36) for the expressed wild type CLR-RAMP1 heterodimer was calculated to be 4.3 ± 0.8μM (*n *= 3). Although the CGRP(1–36) maximal response was similar, this EC_50 _was over 2,600-fold less than the value calculated for CGRP, indicating the importance of the peptide C-terminus F37 for receptor docking. Furthermore, increasing amounts of CGRP(1–36) were also used on HEK293T-RAMP1 cells transiently transfected with L24A, L34A or the L24A,L34A CLR double mutant (Fig 4B). Potency values of CGRP(1–36) to generated cAMP in these transfected cells were 3.4 ± 0.7μM (L24A), 5.6 ± 0.8μM (L34A) and 4.2 ± 1.9μM (L24A,L34A), respectively (*n *= 3). These CGRP(1–36) EC_50 _values for leucine CLR mutants were no different when compared to the potency of this modified ligand for increasing cAMP through the wild type receptor. Moreover, preincubation with 1μM of the CGRP receptor antagonist, CGRP(8–37), abolished the activity of 10μM CGRP(1–36) to generate cAMP through the wild type and leucine CLR mutants, indicating that this response was receptor specific (Fig 4B).

To substantiate results using CGRP(1–36), we characterized another truncated CGRP receptor active peptide, CGRP(1–19), which still lacked a C-terminal phenylalanine. Increasing amounts of CGRP(1–19) were able to generate increasing amounts of cAMP in HEK293T-RAMP1 cells transiently expressing the wild type CLR in a concentration-dependent manner (Fig 4C). The EC_50 _of CGRP(1–19) for the expressed wild type CGRP receptor was calculated to be 2.1 ± 0.6μM (*n *= 3). This EC_50 _value was similar to the potency of CGRP(1–36), but was considerably less than the calculated value of CGRP for the wild type receptor. This potency loss substantiates previous findings that illustrate the importance of this C-terminal CGRP-F37 for receptor docking. Furthermore, the maximal response initiated by CGRP(1–19) was appreciably less when compared to amounts of cAMP generated by activating the wild type receptor using CGRP(1–36) or the endogenous neuropeptide. This finding points to the contribution of peptide residues linking the C-terminal operative domains of CGRP for receptor agonism. Similarly, increasing amounts of CGRP(1–19) were also used on HEK293T-RAMP1 cells transiently transfected with L24A, L34A or the L24A,L34A CLR double mutant (Fig 4C). Potency values of CGRP(1–19) to generated cAMP in these transfected cells were 2.4 ± 0.2μM (L24A), 2.2 ± 0.6μM (L34A) and 1.6 ± 0.2μM (L24A,L34A), respectively (*n *= 3). EC_50 _values of CGRP(1–19) for these leucine mutants are no different when compared to the potency of this modified ligand to increase cAMP through the expressed wild type receptor. The consistent loss of potency for CGRP receptor active ligands lacking a C-terminal phenylalanine compared to the endogenous peptide support previous findings that highlight the importance of CGRP-F37 for docking but not activating the mature receptor and suggests that CRL residues L24 and L34 maybe required for this ligand-receptor interaction.

## Discussion

In this study we hypothesized that specific hydrophobic amino acids from a previously characterized N-terminal CLR deletion mutation [26] are important binding contacts for the C-terminus phenylalaninamide of the endogenous neuropeptide. This premise is based upon earlier structure-function investigations that divided CGRP into two distinct domains based upon its binding and agonistic properties for the endogenous receptor [9]. Initial screening of all hydrophobic residues in this N-terminal domain of the CLR revealed that two leucine residues (L24 and L34), when mutated to alanine, demonstrated a significant binding affinity loss of [^125^I-Tyr]CGRP(8–37), CGRP or AcCGRP(19–37) for these specific CLR mutants when compared to the wild type receptor (Table 1). Differences in the magnitude of binding loss between CGRP and AcCGRP(19–37) for the wild type versus mutant CLRs can be attributed to the interaction of CGRP residues 8–18, which have been shown to also contribute towards the high affinity binding value of the ligand-receptor interaction [18,30]. Moreover, there were no additional changes in the binding deficiency for [^125^I-Tyr]CGRP(8–37), CGRP and AcCGRP(19–37) when both leucine mutations were expressed in the same receptor protein, indicating that there is a cooperative but not synergistic relationship between these CLR residues for binding CGRP-F37 ligands. Additionally, the C-terminal phenylalaninamide is a shared residue between [^125^I-Tyr]CGRP(8–37), CGRP and AcCGRP(19–37). Therefore, compared to wild type CLR, binding affinity losses of these peptide ligands for the leucine CLR mutations suggests that L24 and L34 are significant binding contacts with CGRP-F37.

To support this hypothesis, we examined the binding properties of truncated CGRP ligands lacking the C-terminal phenylalanine for the wild type and leucine CLR mutations. There was a significant binding affinity loss of CGRP(1–36) or CGRP(1–19) when compared to the endogenous neuropeptide for the wild type CLR. These differences were not just due to modifications of functional groups at the carboxyl end because CGRP(1–36) was synthesized with an amidated C-terminus. Furthermore, this dissimilarity between CGRP(1–36) and CGRP was not due to configuration variations because previous biophysical measurements using circular dichroism spectroscopy and nuclear magnetic resonance have demonstrated no significant differences in the secondary structure of these peptides [16]. This deficiency in binding affinity can be directly related to loss of the C-terminal phenylalanine, as has been described by others [15]. However, this affinity loss was no different from wild type receptor when CGRP(1–36) or CGRP(1–19) were used to compete for specific L24A, L34A or L24A,L34A mutant CLR binding sites on transiently transfected HEK293T-RAMP1 cells. No changes in the binding parameters of these truncated C-terminus CGRP ligands for mutant or wild type CLRs indicates that similar contacts for peptide docking are shared between these membrane proteins. This is contrasted with binding affinity differences of CGRP ligands having a C-terminal phenylalaninamide for leucine CLR mutations versus wild type receptor. The only variation between these two sets of experiments is the C-terminus phenylalanine found on the endogenous neuropeptide. This binding property difference of truncated C-terminus peptide ligands versus CGRP for leucine mutants and wild type receptor proteins, strongly advocates CLR L24 and L34 as important binding contacts for CGRP-F37. Moreover, this is the first description for identifiable binding contacts on the CLR that specifically interacts with this essential neuropeptide phenylalaninamide.

We also assessed the functionality of these leucine CLR mutants to increase the CGRP mediated production of cAMP in an effort to understand the importance of these receptor residues for agonist-dependent activation. When compared to wild type receptor the potency of CGRP to increase cAMP production was no different for all three CLR leucine mutations (*i.e*., L24A, L34A and L24A,L34A; see Table 2). This is contrary to the 6- to 8-fold loss of CGRP binding affinity for these same CLR mutations. The ability of CGRP to activate both mutant and wild type receptors with similar potencies suggests there is some plasticity in the agonist binding pocket that can overcome variations in how the peptide docks with the receptor. As figure 1 illustrates, changes in docking the C-terminal portion of CGRP (*i.e*., F37) to these leucine CLR mutants, may alter presentation of the N-terminus CGRP domain to the agonist binding pocket of the mature receptor. However, no changes in the potency of CGRP for the CLR mutants versus wild type receptor indicates there is some flexibility of the CLR agonist binding pocket that can overcome modified docking arrangements of the neuropeptide. Moreover, this explanation for changes in CGRP binding affinity with no differences in potency for mutant versus wild type CLR supports the concept of two distinct neuropeptide domains responsible for receptor binding and agonism [31].

To validate this concept, we tested the ability of truncated C-terminal CGRP ligands to increase generation of cAMP in HEK293T-RAMP1 cells transiently expressing wild type and leucine CLR mutations. If these modified CGRP ligands still retain the essential agonist structures needed for receptor activation, then a concentration-dependent increase in the amount of cAMP generated should be observed even though a necessary amino acid for ligand-receptor docking is missing. For both CGRP(1–36) or CGRP(1–19) there was a concentration-dependent increase in the amount of cAMP produced from HEK293T-RAMP1 cells transiently expressing the wild type CLR (Table 2). However, the potency of these modified ligands was significantly less when compared to CGRP mediated production of cAMP from the same wild type receptor, which is directly related to loss of the phenylalaninamide. These results are similar to investigations where CGRP(1–36) was used for activating endogenous CGRP receptors on acutely isolated pancreatic acinar cells, to increase amylase release [32]. Conversely, there was no significant potency differences of CGRP(1–36) or CGRP(1–19) for increasing cAMP in HEK293T-RAMP1 cells transiently expressing any of the leucine CLR mutations when compared to wild type receptor (Table 2). In addition, this increased cAMP production was specific for activating wild type or mutant CLRs because the response was blocked to basal levels using an effective concentration of the receptor antagonist, CGRP(8–37). The considerable binding affinity loss of truncated C-terminal CGRP ligands for wild type or mutant CLRs, while still being able to activate these same membrane proteins, validates the concept put forth in this study and by others for two distinct peptide ligand domains that manage docking and agonism of the endogenous neuropeptide [31].

Compared to wild type receptor, 8- to 20-fold differences in K_i _values of these leucine CLR mutants for CGRP and AcCGRP(19–37) are not as extensive when contrasted to affinity changes of CGRP(1–36) and CGRP(1–19) for the wild type receptor (Table 1). Absence of these leucine CLR residues cannot explain the entire binding affinity loss of CGRP(1–36) for the wild type receptor. Consequently, it is reasonable to assume that interactions of CGRP-F37 with the mature CLR-RAMP1 heterodimer would necessitate a more complex relationship in order to account for the high affinity binding associated with the loss of this C-terminal peptide residue. For example, the non-peptide CGRP receptor antagonist, BIBN4096BS, has been shown to exhibit high affinity for the endogenous CGRP receptor expressed on SK-N-MC human neuroblastoma cells [33]. In addition, this small molecule CGRP receptor antagonist can selectively be displaced in competition binding assays by CGRP and the receptor antagonist, CGRP(8–37) [34]. Moreover, site-directed mutagenesis studies have documented the importance of a specific extracellular RAMP1 residue in determining BIBN4096BS antagonist affinity for the mature CGRP receptor complex [35]. These observations not only indicate the specificity of BIBN4096BS for the mature CLR-RAMP1 heterodimer but suggest that important high-affinity binding contacts on the mature receptor complex maybe common between BIBN4096BS and CGRP. Therefore, it is plausible that the complex high-affinity receptor binding contacts for CGRP-F37 include not only leucine 24 and 34 on the N-terminal domain of the CLR, but also extracellular residues of RAMP1.

**Figure 5:**
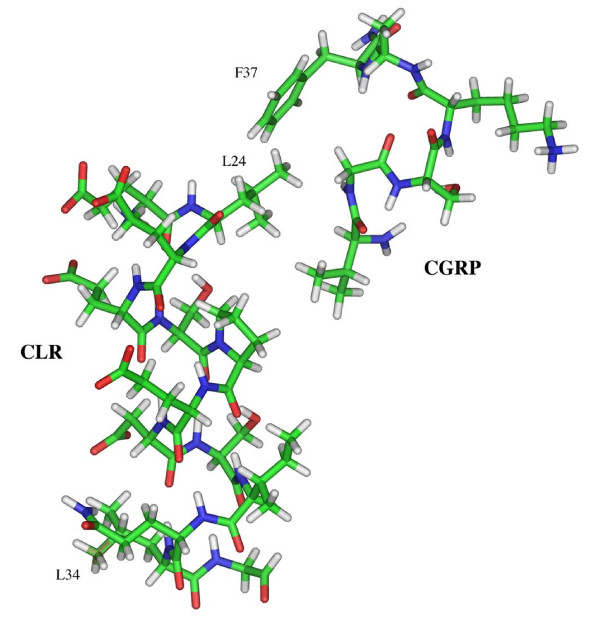
**Working molecular model of CGRP phenylalaninamide interaction with CLR residues L24 and L34**. Energy minimized models for CGRP amino acids 32–37 (NH_2_-VGSKAF-CONH_2_) and CLR amino acids 23–35 (NH_2_-ELEESPEDSIQLG-COOH) were performed as described in the "Methods". Hydrophobic interactions of CLR L24 and L34 with CGRP F37 and RAMP1 (not shown) cooperatively contributes to formation of the high affinity binding pocket essential for docking the C-terminal phenylalaninamide of the neuropeptide with the mature CGRP receptor heterodimer.

To address this idea, a minimized molecular model of a 13 amino acid CLR N-terminal domain fragment interacting with CGRP-F37 is put forward in figure 5. The α-helical structure of this receptor fragment puts L24 on the opposite side of the helix from L34. We believe this minimized secondary structure to be valid because a net negative charge is aligned with a specific face of this α-helix, adjacent to L34. Interestingly, this same N-terminal receptor domain has recently been demonstrated to mediate structural interactions with RAMP1 [36]. Moreover, examination of the RAMP1 extracellular sequence reveals a string of positively charged amino acids adjoining a non-polar hydrophobic region of this accessory protein. We propose that L34 and the adjacent negatively charged helical face of the CLR N-terminus domain interacts with this extracellular region of RAMP1 to form a complex high affinity binding pocket that includes L24 of the receptor. Docking of CGRP-F37 would be accommodated by CLR L24 and other residues formed by the L34 CLR-RAMP1 high affinity binding pocket. A deficiency of either CLR L24 or L34 would cooperatively alter this high affinity binding pocket leading to loss of binding affinity for any CGRP receptor active ligand that contained a C-terminus phenylalaninamide.

In summary, we have characterized specific leucine residues on the CLR membrane protein that participate in the high affinity CGRP binding mediated by the C-terminal phenylalaninamide of the neuropeptide. These CLR leucine residues do not directly participate in CGRP mediated receptor activation because they are not interacting with residues identified with the "agonist" domain of the peptide ligand. Alteration of either leucine CLR residue accounts for a significant loss of CGRP affinity. In addition, this CGRP affinity loss is no different when both leucines are modified in the same receptor protein, indicating that there is not a synergistic relationship between these CLR residues for CGRP binding. This paradigm for distinct binding and activation domains on CGRP for the mature receptor, as illustrated in figure 1, has also been shown using biophysical measurements of the binding mechanism for another group B GPCR [37]. Although lacking the potential binding contributions of RAMP1, which are currently being investigated, this study is the first description of identifiable CLR residues important for docking CGRP to the receptor and will be useful in future studies that would build on our knowledge of this molecular relationship.

## Conclusion

This study is the first to describe specific amino acids on the CLR N-terminus domain postulated to selectively interact with the C-terminal phenylalaninamide of CGRP, previously shown to impart the high affinity relationship of the neuropeptide with the mature CGRP receptor. This deduction is centered upon pharmacological properties of site-directed CLR mutations that are analogous to previous structure-function properties of the endogenous neuropeptide. Based on results from this study, a fundamental model of CGRP interaction with these N-terminus CLR leucine residues is brought forward. This molecular model serves as a starting point for future studies that examine the relationship of the CLR in forming a critical high affinity binding pocket with RAMP1 for CGRP. Our increased knowledge of the mature CGRP receptor binding pocket could lead to development of selective non-peptide small molecule ligands that would be useful in dissecting the important physiology of this abundant neuropeptide correlated with the pathological mechanisms for many vascular related diseases.

## Methods

### Materials

Wild type human CLR clone (GenBank:NM 005795) was a gift kindly provided by Steven M. Foord (GlaxoSmithKline Medicine Research Centre). Amidated CGRP(1–36) was custom synthesized by The Cleveland Clinic Foundation, Lerner Research Institute Core Peptide facility (Cleveland, OH). CGRP, CGRP(8–37), CGRP(1–19) and AcCGRP(19–37) was purchased from American Peptides (Sunnyvale, CA). Tyr^0^-CGRP(8–37) was a gift from Phoenix Pharmaceuticals (Belmont, CA). All peptides used for this study were amidated at the peptide C-terminus except CGRP(1–19). [^125^I-Tyr]CGRP(8–37) was synthesized by Robert C. Speth at the University of Mississippi Peptide Radioiodination Service Center (University, MS). Dulbecco's minimum essential medium (DMEM) and fetal bovine serum (FBS) was purchased from Atlanta Biologicals (Atlanta, GA). HEK293T stably transfected with RAMP1 was a gift kindly provided by Michel Bouvier (University of Montreal, Quebec, Canada). cAMP Biotrak enzyme-linked immunoassay was purchased from Amersham Biosciences (Piscataway, NJ). Benzamidine, leupeptin, and phenylmethysulfonylfluoride was purchased from Sigma-Alrich (St Louis, MO). Whatman GF/C filter paper was purchased from Brandel (Gaithersburg, MD). All other acids, bases and salts were of the highest purity commercially available.

### Site-directed mutagenesis

Site-directed mutagenesis was performed on a pcDNA3.1 human CLR construct utilizing QuickChange site-directed mutagenesis kit (Stratagene, La Jolla, CA) according to the manufactures instructions. Transformed plasmid DNA from bacterial colonies that grew on selective media were isolated (Qiagen, Valencia, CA) and sequenced by the dideoxy method to verify the correct CLR mutation [38].

### Mammalian cell culture

The parent and stably transfected HEK293 cell lines have previously been shown not to express endogenous calcitonin receptor or CLR proteins [22,39,40]. CLR negative HEK293T cells stably transfected with RAMP1 were propagated in DMEM plus 10% FBS under standard cell culture conditions. Confluent HEK293T-RAMP1 cells were washed in Hank's Balanced Salt Solution (HBSS), trypsinized and seeded at the appropriate density in 35 mm 6-well plates or P-150 dishes to ensure 50–80% cell confluence within 24 h. Transient transfection was performed after 24 h with GeneJammer transfection reagent according to the manufactures protocol (Stratagene, La Jolla, CA) using a 3μL reagent per 2μg plasmid DNA ratio for the transfection cocktail. Crude membrane preparations or cAMP signaling assays were performed on these HEK293T-RAMP1 cells 60–72 h post-transfection.

### Crude membrane preparation

A crude cell membrane preparation was prepared as previously described [41]. Briefly, HEK293T-RAMP1 membranes were prepared by scraping and transferring cells to a 50 mL conical tube using cold HBSS followed with two washings by centrifugation at 1000 × g using cold HBSS. The intact cell pellet was resuspended in 10 mL of 0.25 M sucrose containing a protease inhibitor cocktail consisting of 10μg/mL benzamidine, 10μg/mL leupeptin, and 20μg/mL phenylmethysulfonylfluoride. The cells were disrupted by freezing followed by Dounce homogenization of the thawed suspension using 20 strokes from a loose fitting (B) pestle. This mixture was then centrifuged at 1260 × g for 5 min at 4°C. Buffer A (20 mM HEPES, pH 7.5, 1.4 mM EGTA, 12.5 mM MgCl_2_, 10μg/mL benzamidine, 10μg/mL leupeptin, and 20μg/mL phenylmethysulfonylfluoride) was added to the supernatant and centrifuged again at 30,000 × g for 15 min at 4°C. The resultant pellet was kept, resuspended in buffer A then centrifuged once more at 30,000 × g for 15 min at 4°C. The final crude membrane pellet was resuspended in buffer A containing 10% glycerol and stored in aliquots at -70°C until used for radioligand binding. Protein concentrations were measured using the method of Bradford [42].

### Radioligand binding

The radioligand binding protocol used for this study was performed as previously described [41]. Briefly, the density of expressed CLR-RAMP1 heterodimers on HEK293T cells was determined by saturation binding experiments using the selective CGRP receptor antagonist [^125^I-Tyr]CGRP(8–37) as the radioligand. Crude HEK293T-RAMP1 cell membranes were allowed to equilibrate in siliconized polypropylene tubes at 37°C with increasing concentrations of [^125^I-Tyr]CGRP(8–37) in a 0.25 mL total volume of buffer A plus 0.1% BSA using 1μM CGRP to determine non-specific binding. Binding was stopped by filtering the membranes though Whatman GF/C glass fiber filters, followed by 5 – 5 mL washes with cold buffer B (20 mM HEPES, pH 7.5, 1.4 mM EGTA, 12.5 mM MgCl_2_) to remove any unbound drug. Amounts of total and non-specific radiolabel bound to cell membranes were calculated from radioactive counts remaining on the glass fiber filters. From the plotted saturation hyperbola, CGRP receptor density (B_max_) and the equilibrium dissociation constant (K_d_) of [^125^I-Tyr]CGRP(8–37) for CLR-RAMP1 heterodimer binding sites were calculated using iterative non-linear regression analysis [43]. Competition binding studies using increasing concentrations of unlabeled CGRP receptor active ligands were performed in the same buffer as the saturation binding experiments. Iterative non-linear regression analysis was again used to determine the concentration of unlabeled ligand that reduced specific [^125^I-Tyr]CGRP(8–37) binding by 50% (IC_50_). These IC_50 _values were used to calculate the equilibrium dissociation constants (K_i_) of competing CGRP ligands for specific CLR-RAMP1 heterodimer binding sites expressed on HEK293T cells using the method of Cheng and Prusoff [28].

### Quantitation of cAMP generation

60–72 h post-transfection, confluent HEK293T-RAMP1 cells were washed with HBSS then treated at 37°C in serum-free DMEM containing 1 mM 1-methyl-3-isobutylxanthine (IBMX) to inhibit phosphodiesterase in the presence or absence of 1μM CGRP(8–37). After 30 min, increasing concentrations of CGRP receptor agonists were added and the cells incubated for an additional 30 min at 37°C. At the end of this period, cells were lysed by 0.1M HCl and collected for determining the amount of cAMP generated using an enzyme-linked immunoassay according to the manufactures protocol (Amersham Biosciences, Piscataway, NJ). Briefly, ^3^H-cAMP added to cell lysates was used to compete with the endogenously produced cAMP for binding to a specific cAMP-binding protein. ^3^H-cAMP levels were then counted by liquid scintillation and related to endogenously generated cAMP by comparison with known standards. The concentration of CGRP receptor agonists that caused a half-maximal generation of cAMP (EC_50_) was calculated using nonlinear regression analysis (Graphpad, San Diego, CA).

### Molecular modeling

The Biopolymer module in the Insight^®^II molecular modeling package (Accelyrs, San Diego, CA) was used to build models of CGRP residues 32–37 (NH_2_-VGSKAF-CONH_2_) and CLR residues 23–35 (NH_2_-ELEESPEDSIQLG-COOH). All charges were assigned for a pH value of 7. After building the CGRP C-terminal hexapeptide as an extended chain, backbone torsion angles of the peptide sequence were modified to match those from the original structure described by Breeze *et al*. [44]. The CLR peptide was built as a helix then subjected to energy minimization with the Discover module of Insight^®^II using the Amber forcefield and a scalar dielectric constant of 78.54 (water at 25°C) [45]. Energy minimization for the CLR peptide was achieved in two stages. The first stage of 500 steps was carried out using the steepest descents algorithm. The second stage was performed using the conjugate gradient algorithm with a convergence criterion of the derivative < 0.001, converging after 1390 iterations.

### Statistical analysis

For each individual experiment, the fitted iterative nonlinear regression curve that best represented the data was determined using a partial *f-*test, *F *= [(SS1-SS2)/SS2]/[(DF1-DF2)/DF2], where SS = sum of the squares and DF = degrees of freedom (*P *< .05). From the best fit curve, EC_50 _and K_i _values were calculated and compared between wild type and mutant CGRP receptors from concomitantly transfected HEK293T-RAMP1 cells. Significance between groups was tested using an unpaired two-tailed Student's *t *test (*P *< .05). All values are reported as the mean ± S.E. for *n *experiments, each performed in duplicate.

## Authors' contributions

SB generated the molecular reagents, carried out the receptor characterization experiments, developed the initial molecular model and originally drafted the manuscript. JE participated in the cell biology studies. EH participated in the cell biology and binding studies. SLL finalized the molecular model and helped to draft the manuscript. KAT coordinated and oversaw the molecular modeling studies and helped to draft the manuscript. JEP conceived, monitored, and coordinated the experimental design and drafted the manuscript. All authors read and approved the final manuscript.
