# Correction: Identification of specific calcitonin-like receptor residues important for calcitonin gene-related peptide high affinity binding

**DOI:** 10.1186/1471-2210-6-14

**Published:** 2006-12-06

**Authors:** Sugato Banerjee, Janel Evanson, Erik Harris, Stephen L Lowe, Robert C Speth, Kathryn A Thomasson, James E Porter

**Affiliations:** 1 Department of Pharmacology, Physiology & Therapeutics, School of Medicine & Health Sciences, University of North Dakota, Grand Forks, ND 58202-9037, USA; 2 Department of Chemistry, University of North Dakota, Grand Forks, ND 58202-9024, USA; 3 Department of Pharmacology, School of Pharmacy, University of Mississippi, University, MS 38677-9802, USA

## Abstract

This is a correction article.

After publication of this work [1], we became aware of the fact that Robert C. Speth was not included as an author. Dr. Speth put a considerable amount of time and effort into developing and preparing the radiopeptide used to carry out the radioligand binding studies reported in this manuscript and therefore should have originally been included as an author. We apologize to Dr. Speth for any inconvenience that this oversight might have caused and thank him for his invaluable contribution to this project.
